# Information processes of task-switching and modality-shifting across development

**DOI:** 10.1371/journal.pone.0198870

**Published:** 2018-06-18

**Authors:** Anna Peng, Natasha Z. Kirkham, Denis Mareschal

**Affiliations:** Department of Psychological Sciences, Birkbeck University of London, London, United Kingdom; Swinburne University of Technology, AUSTRALIA

## Abstract

Developmental research on flexible attentional control in young children has often focused on the role of attention in task-switching in a unimodal context. In real life, children must master the art of switching attention not only between task demands, but also between sensory modalities. Previous study has shown that young children can be efficient at switching between unimodal tasks when the situation allows, incurring no greater task-switching costs than adults. However, young children may still experience a greater demand to shift attention between modalities than older participants. To address this, we tested 4-year-olds, 6-year-olds and adults on a novel cross-modal task-switching paradigm involving multisensory detection tasks. While we found age differences in absolute reaction time and accuracy, young children and adults both exhibited strikingly similar effects in task-switching, modality-shifting, and the interaction between them. Young children did not exhibit a greater attentional bottleneck on either the task level, or on the modality level; thus, the evidence suggests that young children engaged in similar cognitive operations in the current cross-modal tasks to adult participants. It appears that cognitive operations in multisensory task configuration are relatively mature between 4 and 6 years old.

## Introduction

Rarely do we get an opportunity to fully absorb ourselves in one task (e.g. reading a book), without the occasional diversion to other unrelated tasks (e.g. being called upon to put dinner plates away). In real life, switching tasks often involves switching the modalities we are attending to at the same time. However, the majority of research on task switching and attentional control has been carried out in a unisensory context [1, 2]. For young children who are just about to enter formal education, the ability to self-regulate attention over reactive processing in a multisensory environment is paramount to their ability to meet the demand of formal schooling [3]. Young children must develop a set of skill that allows them to both attend to the task at hand (e.g. reading a worksheet), and paying attention to instructions given by the teacher, while resisting distraction from their peers.

Flexible attention control is often looked at using task-switching methods, but there are wide variations between developmental studies with young children and adult cognitive studies. The paradigms used with adult participants generally involve switching between relatively simple cognitive tasks, such as identifying either a target form or a target color in a sequence of stimuli [4–8]. These studies have reported a reliable cost in reaction time (RT) when switching to another task, as compared to repeating the same task. In developmental studies with young children, the experiments often involve a condition whereby the children develop a prepotent representation of the stimulus (e.g. the form), and the core interest is how children overcome the strong but irrelevant representation and shift their attention to the alternative attribute of the stimulus required by the second task.

While both adult and developmental studies are motivated by their interest in cognitive and attentional flexibility, majority of these studies involve visual or auditory tasks only. It therefore remains unclear whether the attentional flexibility abilities involved in switching between tasks also subserve the ability to shift attention between *sensory modalities*, and whether both abilities undergo similar development in the early childhood.

We already know that the development of cognitive and attentional control is long and protracted [9]. In comparison, perceptual and multisensory systems show rapid progression in both differentiation and emergent interactions by the age of 2 [10, 11]. Although multisensory system is already in place at an early age, children still exhibit many subtle differences in how they respond to multisensory information as compared to adults, such as the differences in modality dominance [12–14], multisensory integration [15, 16], cross-modal interference [17, 18] and multisensory learning [19]. It is possible that the cortical maturation that underpins cognitive and attentional development, also implicates multisensory development, given that attentional control may have a direct effect on how cross-modal information is processed [20].

Thus, the present study aims to identify and understand any developmental differences in information processing in cross-modal task-switching that might exist between young children and adults. In what follows, we first look at the limited research on cross-modal task-switching in the adult literature, then give a brief overview on attentional flexibility and cross-modal interaction in developmental literature, before outlining our study and hypotheses.

### Cross-modal task-switching

Early studies using dual visual and auditory detection tasks suggested that visual and auditory processing are largely distinct [21]. However, shifting attention to another modality results in reliable RT costs [22], implying that cross-modal attentional shifts tap into a common processing resource shared between modalities. An alternative explanation is that this modality shift cost is largely attributable to task-switching costs [23], since when the participants shift attention cross-modally, they also attended to an alternative target. One critical question, therefore, is whether the presentation modality is just another task attribute like color and shapes in unisensory task-switching experiments. One way to answer this question is to look at the interaction between different types of attentional transitions (modality vs. task) and response transitions. Past research on task-switching has reported that response repetition is facilitative only when the task repeats, but not when the task switches [7]. Indeed, Cohen and Rist [24] found that modality shift effect was only evident when a response was executed previously. However, without a well-defined task that bridge across the auditory and visual stimuli, any modality associated effect observed may be contaminated with task associated effect.

To date, there have been only a handful of cross-modal task-switching studies in which both visual and auditory stimuli shared the same task attribute [25–27]. As with the usual unisensory task-switching procedure, Hunt and Kingston [25] asked the participants switched between two simple number choice tasks (odd/even vs. large/small), with a critical modification where the stimulus could be either visual or auditory. They found that task-switching and modality-shifting each produced reliable RT costs. While the reaction time on the trials requiring a simultaneous modality and task change (modality-shift task-switch trials, or MSTS) was the longest, the observed cost was less than the combined task-switching and modality-shifting costs when added together individually. In other words, a *subadditive* effect to the combined costs was observed. One explanation for this finding is that modality-shifting and task-switching are both independent but also interdependent, subjected to a common processing bottleneck yet facilitated through separable pathways. In contrast, Murray et al. [26] found a stronger *facilitative* effect of modality-shifting on task-switching, as RT on MSTS was faster than on trials with task-switch alone (i.e. with modality repetition). Their experiment involved where/what classifications on either man-made or natural objects/sounds. They explained the result through both interference and an episodic binding effect between task and modality, such that when one index changes (e.g. modality), there is a relative advantage when the task changes as well due to relatively reduced interference in the network.

Both Hunt and Kingston and Murray et al. employed unimodal stimuli in their studies, but a similar effect can also be found with bimodal stimuli. For example, Sandhu and Dyson [27] employed different cueing methods in their study (no-cue, single-cue to either modality or task, and dual-cue to both modality and task) and found a general subadditive effect on trials with simultaneous modality and task change, although the effect was not as large as that reported in Murray et al.’s study [26]. They also found some differences between modality-shifting and task-switching. Modality shift costs in RT were only evident in the no-cue condition, whereas task switch costs in RT were reliable in both no-cue and single-cue conditions. Thus, it appears that task representations are more complex and only when full information is provided would the processing costs be effectively eliminated. The result suggested that task-switching and modality-shifting may engage different attentional operations, and that modality-shifting appears to be less cognitively taxing than task-switching. However, in both cases, RT costs were eliminated when both modality and task cues were provided.

In contrast to the results from cross-modal task-switching studies, a complete elimination of processing costs in unisensory task-switching experiments was rarely reported, even with univalent stimuli where only one task was possible on each trial [28–30]. It is therefore important to understand how simultaneous change in task and modality reduces and/or eliminates processing costs.

### Attentional flexibility and multisensory development

Children between the ages of 3 and 6 experience considerable change in cognitive and attentional control [1, 9, 31]. In particular, they show dramatic improvements in cognitive flexibility and in the ability to manipulate multiple mental representations, shift goals and moderate their responses [9, 32]. By age 6, children are typically able to perform tasks that require them to shift attention between different attributes of a stimulus, with improvements in this ability continuing into late childhood [33]. Another significant change during this period is the reduction in perseverative errors [34–36] Because the performance is typically measured in pass/fail, developmental change can look abrupt with 3-year-olds failing and 5- to 6-year-olds passing the tasks. This may mask the continuity of development across early childhood. Reaction time provides a continuous measure of development; however, with young children, there are fewer task-switching studies using reaction times as a dependent measure than using pass/fail error criteria, and those that do exist often report conflicting findings. Within the developmental studies that measured reaction time, some have reported an age effect on task-switching associated costs [37–42], whereas others have either found only a small or no age effect[43–46]. In at least one case, though, young adults were found to show a larger RT switch cost than older children [45].

In fact, whether an age effect on switch associated RT costs is observed or not appears to be very dependent on task parameters such as task cues, the duration of the response window, levels or types of task conflicts, and the task rules. Moreover, in studies that involved young children, those that observed a stronger developmental effect have also tended to report a much lower overall accuracy among the youngest children (e.g. [35, 37]). Consequently, it is unclear whether task-switching costs in RT and accuracy reflect young children’s general inability to switch between goal representations, or, if young children are affected by other parameters specific to the individual task [44]. Indeed, young children and older participants may perceive stimulus and response sets qualitatively differently. For example, children aged between 5 to 7 years of age experienced greater interference from stimulus attributes, but not from response overlaps; whereas children older than 9 experienced greater interferences from response overlaps but not from stimulus attributes [38,47].

It follows that if the stimulus categories and response sets are familiar to both children and older participants, and the level of task conflicts are age-appropriate, even young children may be able to shift goal representations flexibly. Our previous work has shown that when the stimulus attribute was easily retrieved (such as an animal category), and the response selection was simple (one-button response), there was no reliable age interaction effect on task-switching costs between children aged 4 and 6, and adults [44]. Children as young as 4 were effective at preparing for the upcoming task given the appropriately familiar context.

Although young children can be as effective at switching between tasks as adults, given an age-appropriate task, they may still exhibit cross-modal task-switching effects different from those of adults due to a developing multisensory system. While there is no direct developmental research on cross-modal task-switching, children and adults show differences in brain activation patterns in cross-modal oddball detection. Johannsen and Röder [48] looked at the refractory effect of within-modal and cross-modal oddball detection tasks. They found that while young children aged between 4 and 6 showed similar overall refractory effects to within-modal target detection, compared to older children aged 10 and 12 and adults, the cross-modal refractory effect was different in the youngest group. Young children showed cross-modal refractory effects earlier in processing as compared to older participants, and the supra-modal interaction of cross-modal information was stronger in young children than older children and adults. Topographically, younger children also exhibited more activation in the frontal region, an area that is heavily implicated in cognitive control, as compared to the parietal topography found among older children and adults. The result suggests that younger children are more likely to exert endogenous influence to direct attention cross-modally, perhaps to overcome for the greater neural interference due to the less specialised (i.e. less segregated) short-range networks [49,50]. If so, children may experience a greater attentional bottleneck in conditions in which cross-modal attentional shifting is required.

### The current study

The current study is designed to explore whether processing costs in RT and accuracy associated with modality-shifting and task-switching reflect any developmental changes. Our study focused on preschool years as many studies have documented a dramatic change in cognitive control during these critical years [1]. The current cross-modal task-switching study employed a simple detection task with a single response key. Using a single response key minimises the demands on response selection, and ensures that the observed response latency reflects the time needed to shift attention at the level of modality and task goal.

We make a number of predictions based on the literature reviewed above. Firstly, consistent with the previous literature, we hypothesise that both task-switching and modality-shifting would produce attentional costs, in both children and adults. Secondly, when a child-friendly procedure is used we hypothesise that young children can be as effective as adults at switching between task goals and selecting the appropriate stimulus attribute to respond on. Thirdly, we expected that children will require more endogenous control than adults in order to shift attention cross-modally. As a result, we predict that younger children will exhibit greater modality shift costs than older children and adults. Finally, if younger children require greater endogenous control to shift attention cross-modally, they will show additive costs of modality-shifting and task-switching, whereas adults will benefit from modality-shifting during task-switching, and therefore show a facilitative effect or at least subadditive costs when switching both task and modality.

## Materials and method

### Ethical statement

Informed parental consent was obtained for each child participant and informed consent from each adult participant in accordance with the University ethics committee guidelines. The research was approved by the Ethics Committee at the Department of Psychological Sciences, Birkbeck University of London (Approval Number: 141556).

### Participants

Forty-two 4-year-olds, 30 6-year-olds and 24 adults were recruited for the experiment. Seventeen 4-year-olds and 4 6-year-olds were excluded from the analyses: 5 4-year-olds and 2 6-year-olds were unable to complete the training element of the study sufficiently well, and 12 4-year-olds and 2 6-year-olds did not pass the baseline accuracy on 70% in the testing session. Consequently, a total of 25 4year-olds (Mean = 4.66 years, SD = 0.23 years, Male = 12), 26 6-year-olds (Mean = 6.51 years, SD = 0.27 years, Male = 17) and 24 adults (Mean = 27.18 years, SD = 8.21 years, Male = 13) were included in the final analyses presented below.

All children were recruited from local primary and nursery schools, with the exception of nine 4-year-olds recruited separately to the testing lab at the research centre. All testing was conducted in a quiet room in the participant’s school or in the lab testing room in the research centre. Children were given token rewards (i.e. stickers) at the end of each block to maintain their motivations. Each session lasted around 25 minutes. Adult participants were recruited from the University campus. No rewards were given to adults and they were tested in a quiet room of the University campus. All participants had normal or corrected to normal vision and hearing.

### Stimuli and design

**Fig 1:**
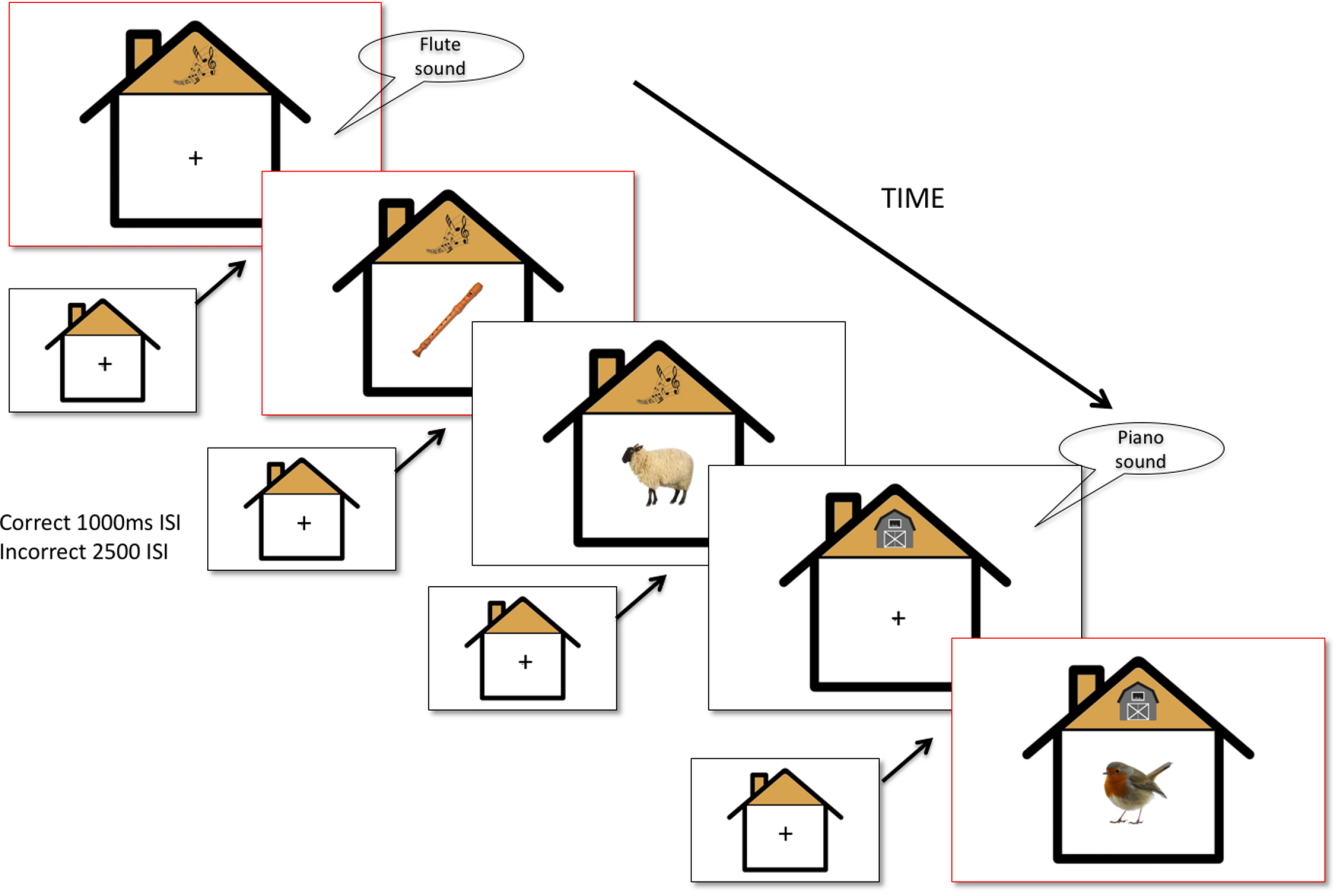
Experimental design. All stimuli were unimodal (either just a sound or just a picture). The experiment involved switching between musical instrument task cued by the musical symbol on the roof, and an animal detection task cued by the barn sign on the roof. ISI (inter-stimulus-interval) was 1000ms for a correct response, and 2500ms for an incorrect response.

The experiment consisted with two different detection tasks: an animal detection task cued by a drawing of a barn house, and a musical instrument detection task cued by a drawing of musical notes. The targets in the animal detection task were various animal pictures and animal sounds, and the targets in the musical instrument task were various pictures of musical instrument and musical sounds. The targets could be presented in either modality and all trials were unimodal (i.e. only a sound or an image was presented). Task cue (approx. 2.2cm x 1.9cm) and the stimulus were presented simultaneously (Fig 1).

There were 10 different colourful pictures (approx. 3.8cm x 3.8cm) and 10 different sounds (approx. 60dB; 42,000 Hz) in each target category (animal vs. musical instrument). The stimulus timed out after 3000ms if no response was made. The target was presented centrally on the screen inside a cartoon drawing of a house (11.4cm x 11.4cm). The task cue was presented above the target as a banner of the house. The inter-stimulus-interval (ISI) was 1000ms for a correct response, or 2500ms for an incorrect response. A continuous background neutral water sound was played at approximately 45dB throughout the experiment, in order to match with the continuous presentation of visual information. The participants received no feedback for the responses.

The experiment involved 120 trials separated into 5 blocks (24 trials per block). Tasks changed unpredictably in each block, with a 60% chance of a correct target appearances (i.e. the number of correct target appeared on 72 trials). Stimulus modality was chosen randomly with an equal probability. The experimental manipulation resulted in a number of cells for four specific trial types: modality-repetition task-repetition (MRTR, Mean = 16.5, SD = 3.7), modality-repetition task-switch (MRTS, Mean = 17, SD = 3.9), modality-shift task-repetition (MSTR, Mean = 19, SD = 2.8), and modality-shift task-switch (MSTS, Mean = 19.5, SD = 3.1).

The experiment was administered on a MacBook Pro Retina with 1440x900 pixels, run on 2.3 GHz Intel Core i7. Child-friendly closed-back headphones were used for presenting the auditory stimuli. The participants made a response by pressing the central spacebar highlighted with a green sticker. Both accuracy and response times were measured.

### Procedure

Children were seated approximately 45cm in front of the laptop. The experiment was divided into four parts—a category session, a demonstration session, a practice session and the testing session. The category, the demonstration, and the practice sessions were conducted with Microsoft PowerPoint. The dependent measures of RT and accuracy were recorded only in the testing session, which was implemented using Matlab R2014b and Psychophysic Toolbox extensions [51–53].

### Categorization, demonstration and practice sessions

The categorization session was designed to make sure that the children understood the categories of the stimuli. There was an auditory categorization task and a visual categorization task, each consisting of 10 trials of pseudorandom presentation of music and animal sounds/pictures. The participants were asked to verbalise whether the sound/picture they were presented with was an animal or a musical instrument. The experimenter made sure that the participant named the category (animal vs. music) and not the actual label of the animal or the musical instrument. The participants who made more than 2 errors in either task were excluded from participating in the test trials used for analyses but were congratulated and given certificates for participating like all other children.

The demonstration and the practice session each consisted of 4 trials of each task (total 8 trials). The first two trials of each task in the demonstration were target trials (one visual target and one auditory target) and the last two trials were non-target trials (one visual non-target and one auditory non-target).

Before the demonstration trials began, children were shown a slide with two houses and were given the instruction as following, ‘we have two different houses here. This house over here is a music hall (pointing to the music hall). I know it is a music hall because it has this symbol on the roof (pointing to the task cue). Can you see the symbol on the roof?’ The experimenter waited for the child to answer. ‘In the music hall, different music is being played. If you find yourself in the music hall, I want you to look for music for me. You might see it in the house, or you might hear it in your ears, but whenever you find music in the music hall, I would like you to tell the computer by pressing the green button (indicating the space bar highlighted with a green sticker).’ A similar instruction was given for the barn house where the participants were instructed to look for animals instead.

The first 4 demonstration trials were examples of the music hall game. A target trial was shown and the experimenter would say ‘Oh here is a music hall (pointing to the cue). We are looking for music.’ The experimenter would pause for approximately 1 to 3 seconds depending on whether it was a visual stimulus or an auditory stimulus. ‘Now tell me, did you see/hear a musical instrument?’ The experimenter would go over the trial again if the participant gave an incorrect answer. After getting the correct affirmative answer, the experimenter told the participant to press the spacebar to let the stimulus into the house if it is a music instrument, or to wait and do nothing it is an animal (participants were also told that if the animals got into the music hall, they would make a mess inside the music hall; and the equivalent story for the ‘barn house’ game was that if music was being played in the barn house, it would wake up the sleeping animals.).

In the practice session, the experimenter told the participant ‘Now I want you to have a little practice before we start the real game.’ When the first trial came up, the experimenter hinted, ‘Oh it is a music hall/ barn house. If you find a musical instrument/ an animal, press the button’. The hints were given on every trial until the experimenter was confident that the participant understood the rule. There were 8 trials in the practice session, and 4 TS (task-switch) trials and 4 MS (modality-shift) trials presented in a predetermined but unpredictable sequence to the participants.

### Testing session

During the testing session, the participants were given trials that were randomised according to task and modality. The experimenter told the participants that they should respond as quickly and as accurately as possible. For the first four trials the experimenter gave verbal reminders to the participants. The verbal reminder was, ‘Here is a music hall/ barn house. If you see/hear music/animal, press the button.’ A total of 120 trials were equally separated into five blocks by a motivational screen offering a rest-break if needed. Children saw the simultaneous presentation of a task cue and the stimulus for a maximum of 4000ms or until a response was made, followed by a variable inter-stimulus-interval (ISI), dependent on the responses—for a correct response, the interval was 1000ms; for an incorrect response, the interval was 2500ms to allow a recovery period (Fig 1). A fixation cross was shown during the ISI. The response window was kept between 300ms and 3800ms after the stimulus onset. The lower bound was designed to exclude anticipatory response and the upper cap was designed to allow for a 200ms gap to process motor feedback (i.e. disappearance of the stimulus and the refreshed screen).

## Results

Both reaction time and accuracy were measured in this study. The between-subject factor was Age and the within-subject factors were Task Transition (Task Switch vs. Repetition), Modality Transition (Modality Shift vs. Repetition), and Response Transition (Response Single vs. Response Repetition). The first trial in each block was excluded from the final data since these trials do not correspond well to a specific trial type (i.e. it is not a switch/repetition of task/modality). Only the correct positive trials were included in the RT analyses. The current study made no assumption about the distribution of the RT samples for each participant. Instead, the bootstrapped mean RTs of each participant were obtained by resampling the RT data for 5000 times thereby ensuring that the data was normally distributed (54). The case for adopting a bootstrap method is particularly valid with the limited RT samples as in the current experiment. The alpha level was set at .05 across all planned comparisons. Unless reported otherwise, all *main effects* of Age in the analyses reported below were significant at at *p*<.05 level.

### Overall accuracy and reaction time

**Fig 2:**
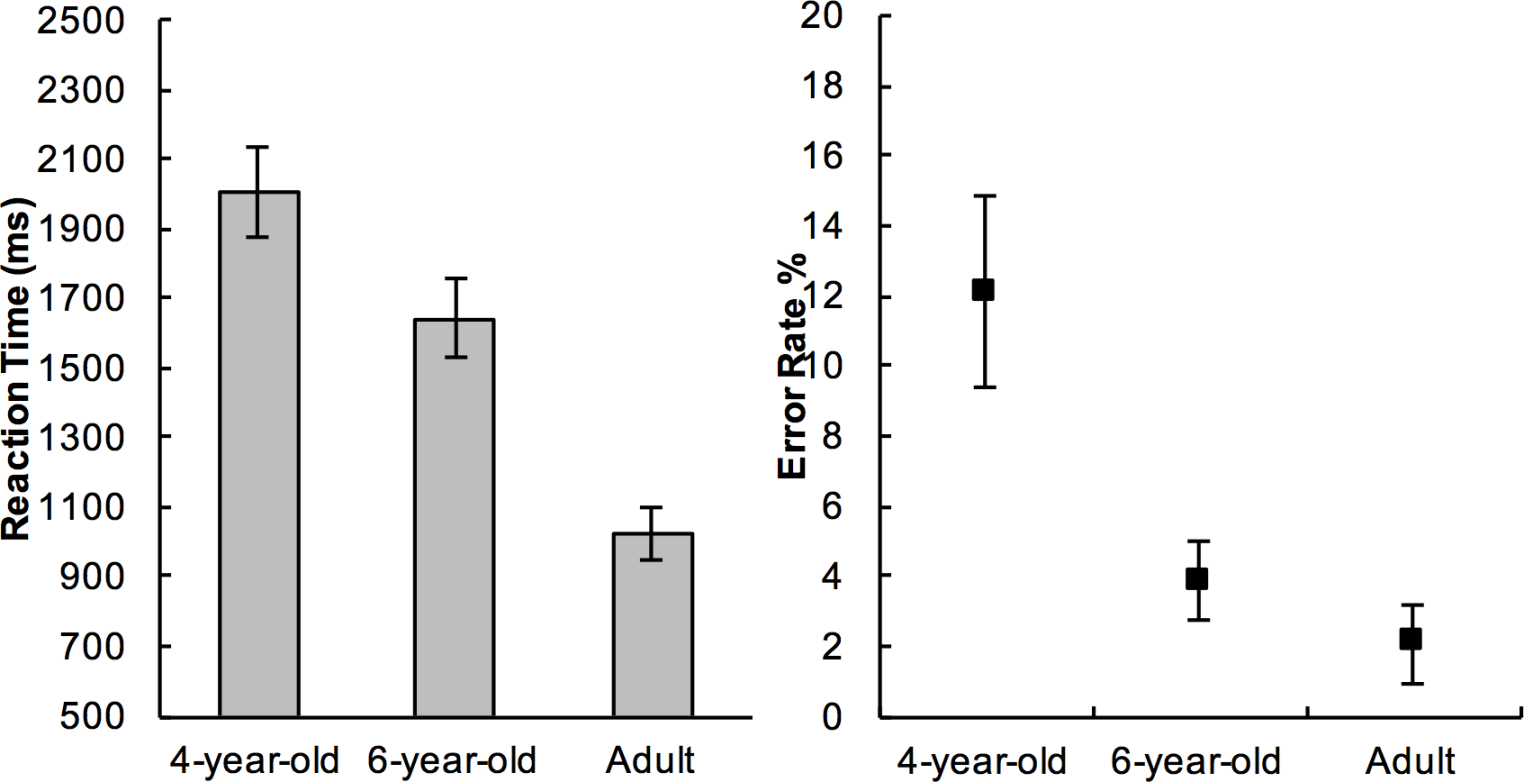
Mean RTs and error rates in each age group. Error bars represent 95% confidence intervals of means.

Fig 2. shows the reaction times (RT) and the accuracy of each age group. All ages achieved high accuracy—4-year-olds (Mean±SE = 88%±1.6), 6-year-olds (Mean±SE = 95.9%±0.5), and adults (Mean±SE = 97.9%±0.7). Younger participants were slower than older participants—4-year-olds (Mean±SE = 2005ms±65), 6-year-olds (Mean±SE = 1595ms±58), adults (Mean±SE = 1020ms±39).

Preliminary analyses using univariate ANOVA were carried out to investigate the between-subject effects of Age on RT and Accuracy. There was a significant effect of Age on both RT (*F*(2,69) = 84.42, *p*<.001, *η*^*2*^=.710) and Accuracy (*F*(2,69)=25.25, *p*<.001, *η*^*2*^=.423). Post-hoc t-tests (Bonferroni corrected to alpha=.017) on RTs showed significant differences among all pairwise comparisons (4-year-old vs. 6-year-old, *t*(49)=4.692, *p*<.001); 6-year-old vs. adults, *t*(48)=8.093, *p*<.001; 4-year-old vs. adult, *t*(47)=12.814, *p*<.001). Only 4-year-olds were significantly less accurate than the older participants (4-year-old vs. 6-year-old, *t*(49)=4.633, p<.001; 4-year-old vs. adult, *t*(47)=5.492, *p*<.001). There was no significant difference in accuracy between 6-year-olds and adults (6-year-old vs. adults, *t*(48)=2.343, *p*=.025, n.s.). Unless reported otherwise, all main effects of Age on RT in the analyses reported below were significant at *p*<.001.

### Target modality

**Fig 3:**
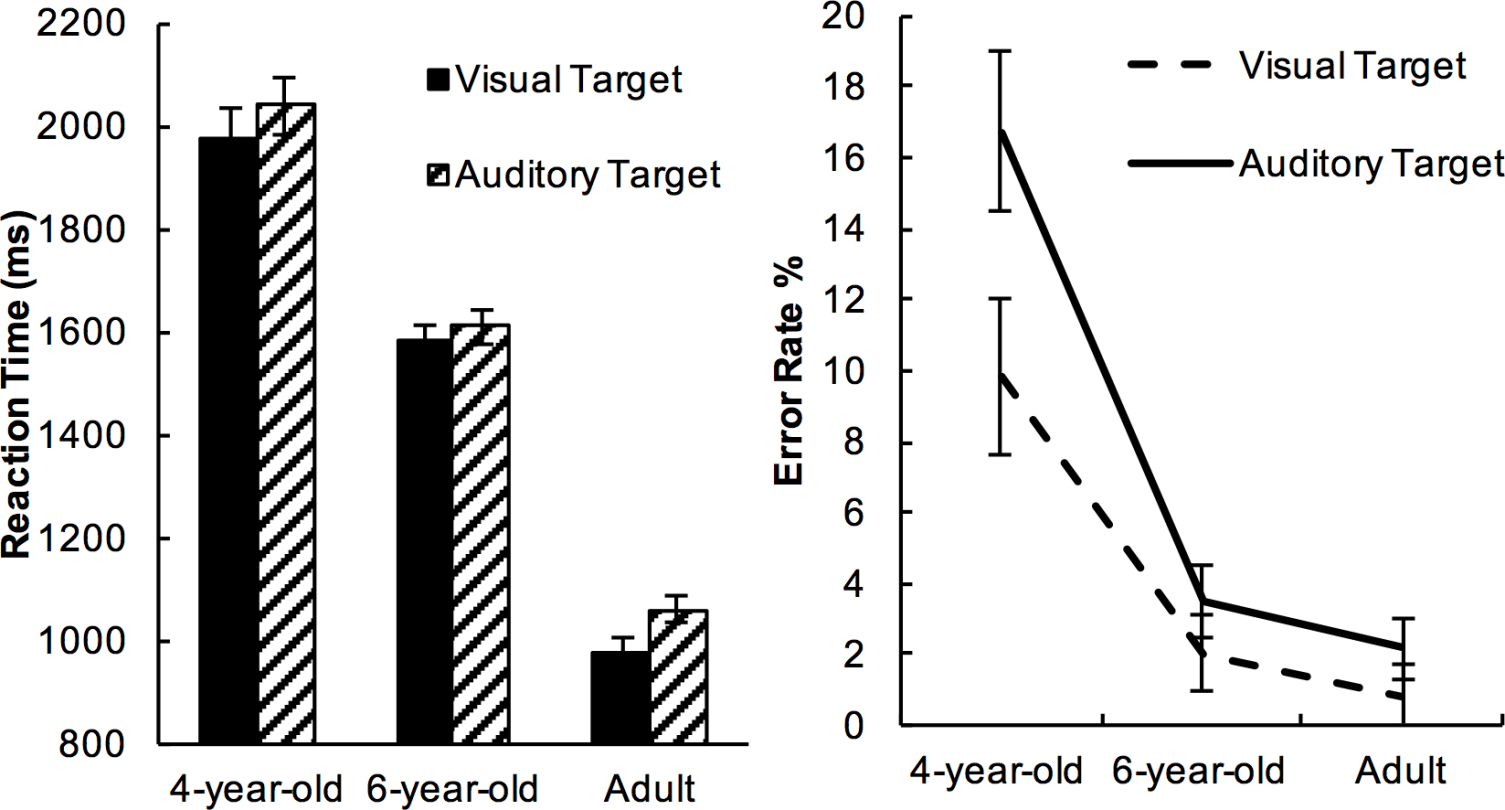
Reaction time and error rate to visual and auditory targets. Error bars represent 95% within-subject confidence intervals of means [55].

Reaction Time. We first looked at whether there were any age differences in how visual and auditory stimuli were processed. Mean values showed that the participants were quicker when responding to visual targets (Mean±SE = 1520ms±58) than to auditory targets (Mean±SE = 1580ms±57) (see Fig 3). Two-way mixed ANOVA was carried out with Target Modality (visual vs. auditory) as the within-subject factor and Age as a between subject factor. The main effect of Target Modality was significant, (*F*(1,72) = 6.52, *p* = .013, *η*^*2*^ = .083) demonstrating that responses to visual targets were quicker than to auditory targets. There was no Target Modality by Age interaction in RT (*p*>.700).

Accuracy. Fig 3. shows that the accuracy was higher for visual targets (Mean±SE = 95.75%±0.88) than auditory targets (Mean±SE = 92.55%±1.3). The main effect of Target Modality was supported in the two-way ANOVA with Target Modality as the within-subject factor and Age as the between-subject factor (*F*(1,72) = 13.15, *p*<.001, *η*^*2*^=.154). The main effect of Target Modality was moderated by an interaction between Target Modality and Age (*F*(2,72)=4.32, *p*=.017, *η*^*2*^=.107). The interaction was followed up with separated analyses within each age group, which showed that only 4-year-olds were significantly less accurate to auditory targets than visual targets (*F*(1, 24)=9.30, *p*=.006, *η*^*2*^=.279), all other groups showed no difference in accuracy between targets of either modality (*ps*>.100).

### Modality-shifting and task-switching

**Fig 4:**
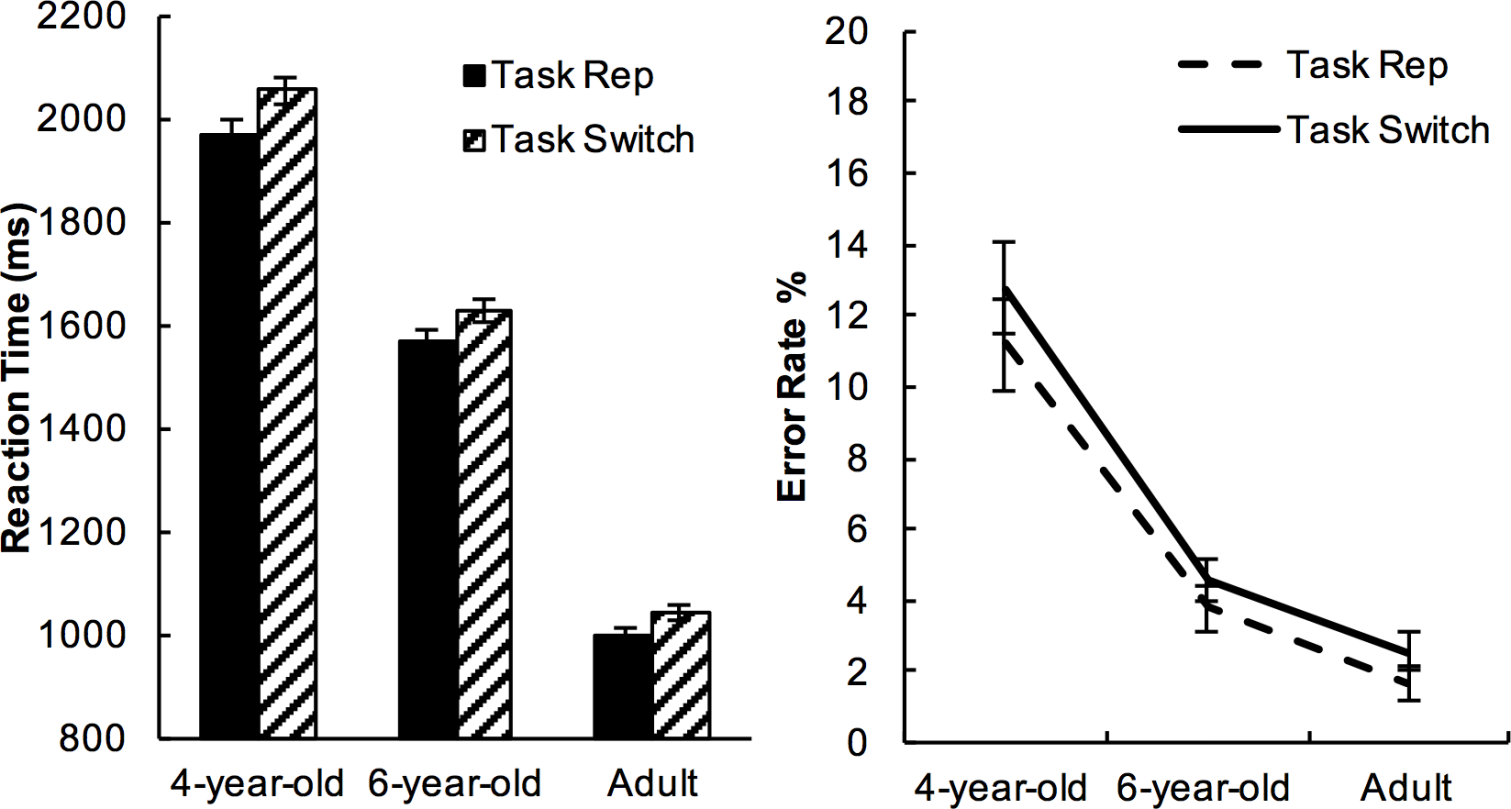
Reaction time and error rates on task-repetition vs. task-switch trials. Error bars represent 95% within-subject confidence intervals of means [55].

**Fig 5:**
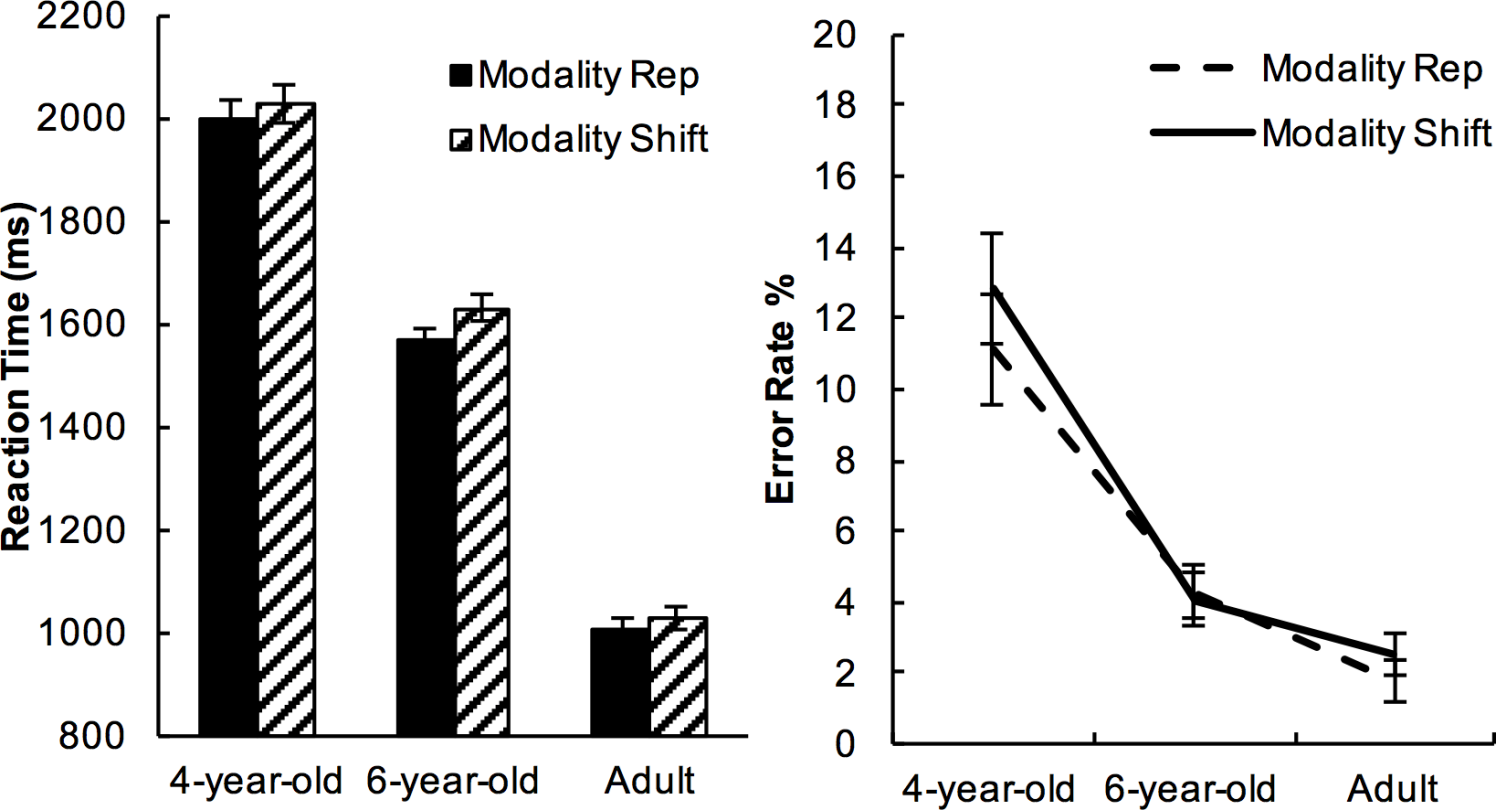
Reaction time and error rates on modality-repetition vs. modality-shift trials. Error bars represent 95% within-subject confidence intervals of means [55].

Reaction time. Mean RTs were longer on TS (task-switch) trials (Mean±SE = 1584ms±58) than TR (task-repetition) trials (Mean±SE = 1519ms±56) (see Fig 4); and longer on MS (modality-switch) trials (1570ms±57) than MR (modality-repetition) trials (Mean±SE = 1532ms±58) (see Fig 5).

The effects of modality-shifting and task-switching were analysed with a three-way mixed ANOVA with Age as the between-subject factor, Modality Transition and Task Transition as within-subject factors. The main effect of Task Transition was significant (*F*(1,72)=25.111, *p*<.001, *η*^*2*^=.259), as well as the main effect of Modality Transition (*F*(1,72)=4.541, *p*=.037, *η*^*2*^=.059). There was no interaction either between the effect of Task Transition and Age (*p*>.400); nor between the effect of Modality Transition and Age on RT (*p*>.400).

We hypothesised that shifting modality would benefit switching task, and expected to find an interaction between Modality Transition and Task Transition. Contrary to our prediction, there was no interaction between Task Transition and Modality Transition on RT (*F*(1,72) = .96, *p*>.300, *η*^*2*^ = .013), nor was there a three-way interaction between Task Transition, Modality Transition and Age (*p*>.300).

Accuracy. Mean accuracy was greater on TR trials (Mean±SE = 94.41%±0.80) than on TS trials (Mean±SE = 93.34%±0.84), and the effect of Task Transition on Accuracy was significant (*F*(1,72) = 4.345, *p* = .041, *η*^*2*^ = .057, see Fig 4). In contrast, the effect of Modality Transition on Accuracy was not significant (p>.200, see Fig 5). There was no further interaction between Task Transition and Modality Transition (*F*(1,72) = 2.30, *p*>.100, *η*^*2*^ = .032), nor a three-way interaction with Age (*p*>.300).

### Cross-modal task-switching (CMTS) cost vs. combined cost

**Fig 6:**
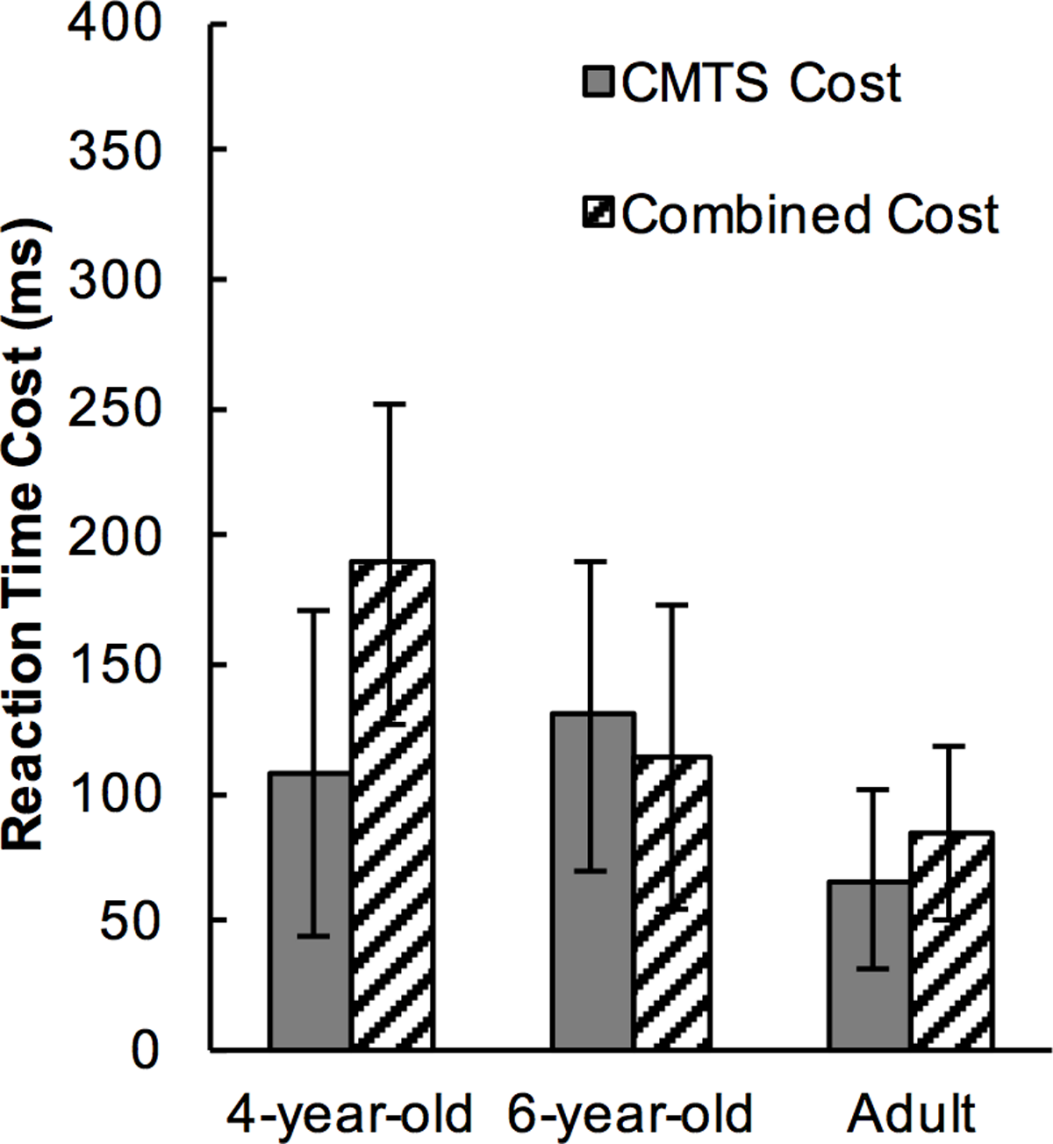
Cross-modal task-switching cost and combined cost in reaction time in different age groups. Error bars represent standard error of means.

While the task-switching effect was similar in both the modality-shift trials and the modality-repetition trials, the effect of CMTS might manifest itself not in terms of a reduction of TS cost, but as a cost subadditive to the combined TS and MS effects. To understand this, we calculated the CMTS cost and the combined cost. The CMTS cost is the RT differences between MSTS trials and MRTR trials. The combined cost is the overall cost of TS cost (RT differences between MRTS trials and MRTR trials) and MS cost (RT differences between MSTR trials and MRTR trials). Mean RTs showed that the CMTS costs and the Combined costs were very similar (Fig 6).

A two-way mixed ANOVA was carried out on Cost Type as the within-subject factor (CMTS cost vs. combined cost) and Age as the between subject factor. The result returned no significant main effect of Cost Type (*p*>.300), nor any interaction with Age (*p*>.300). The overall result failed to find any facilitative effect of CMTS on task-switching, or as a subadditive cost to the combined TS and MS costs.

### The effect of response repetition on task-switching and modality-shifting

**Fig 7:**
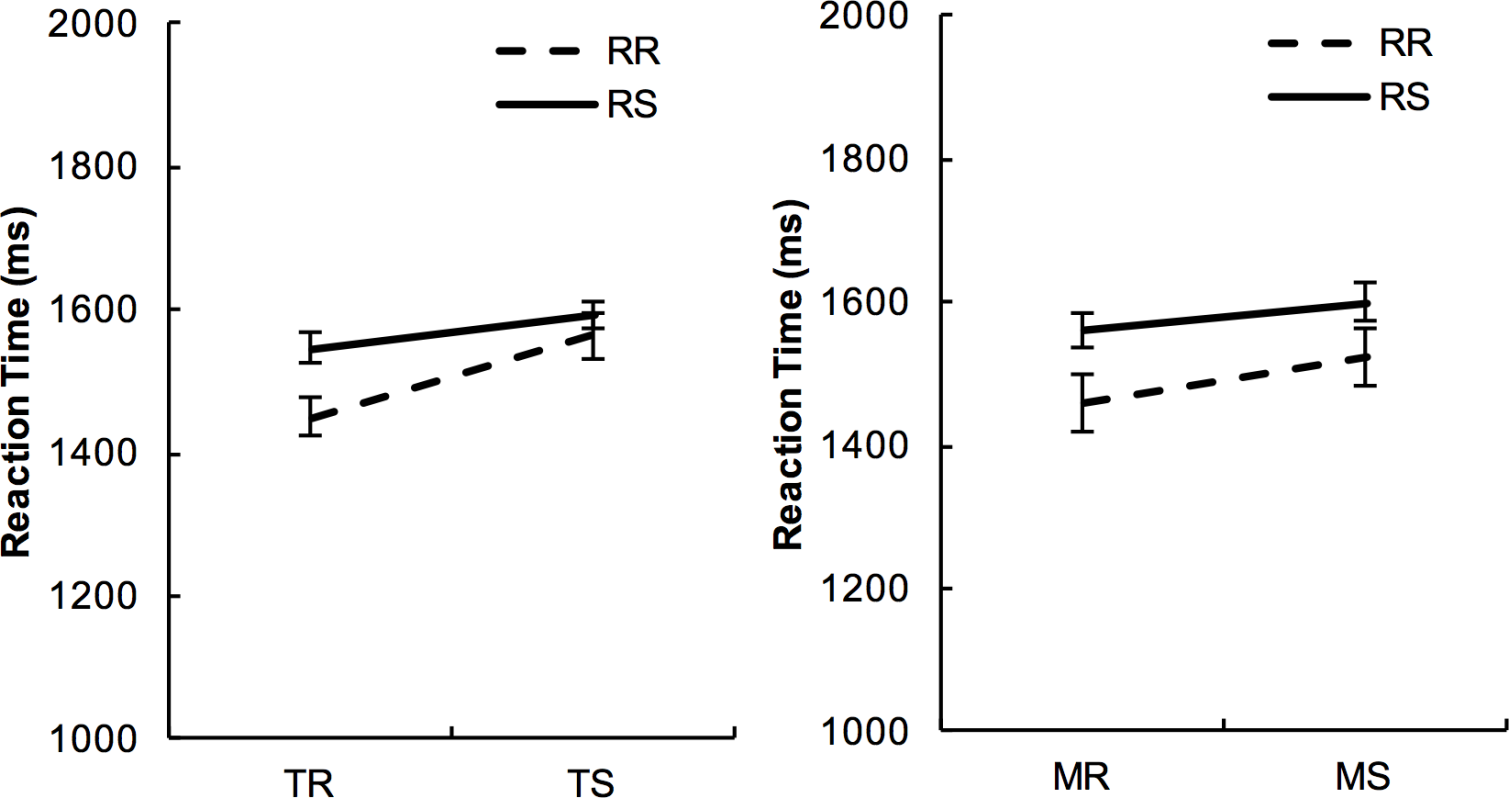
Response repetition (RR) effect under different attentional transition. Left panel shows RR effect and Task Transition; right panel shows RR effect and Modality Transition. RR: Response-Repetition; RS: Response-Single. Error bars represent 95% within-subject confidence intervals of means [55].

Mean RTs indicated that the participants were quicker at responding when the trial was preceded by a response (Response Repetition, RR) than when it was not preceded by a response (Response Single, RS). Fig 7 shows the effect of Response Transition in different Trial Types (task-repetition, task-switch; modality-repetition, modality-shift).

The effect of Response Transition was analysed with the respect of Task Transition with a three-way mixed ANOVA (within-subject factors: Response Transition and Task Transition, 2 levels each; between-subject factor: Age). Only correct responses were analysed. The mixed model showed that there was a significant main effect of Response Transition (*F*(1,72)=15.899, *p*<.001, *η*^*2*^=.181), which was superseded by a significant higher-order interaction between Response Transition and Task Transition (*F*(2,72)=5.28, *p*<.024, *η*^*2*^=.068). There was no further interaction with Age (*p*>.400).

The interaction between Response Transition and Task Transitions were followed up by analysing the effect of Response Transition on TS trials and TR trials separately, with all ages collapsed together. The effect of Response Transition was only evident on the TR trials (*F*(1,74)=19.83, *p*<.001, *η*^*2*^=.211), but not on TS trials (*F*(1,74)=1.81, *p*=.182). The interaction indicates that when the task repeated, responses were faster on the RR trials (Mean±SE=1451ms±56) than on the RS trials (Mean±SE=1545ms±57); in contrast, when the task switched, response times were similar on the RR trials (Mean±SE=1564ms±60) and on the RS trials (Mean±SE=1592ms±58). The result is consistent with the past literature that response repetition is only facilitative when the task repeats, but not when the task switches.

Similarly, Response Transition was also investigated with the respect of Modality Transition (Response Transition and Modality Transition were entered as within-subject factors, and Age as a between-subject factor), on RTs of correct responses. While the main effect of Response Transition remained significant (*p*<.001), in contrast to the interaction between Task Transition and Response Transition, there was no two-way interaction between Response Transition and Modality Transition (*p*>.500). This demonstrates that responses were faster on the RR trials than on the RS trials irrespective to modality transition. There was no further interaction with Age (*p*>.200).

## Discussion

Using a novel task-switching procedure that involved unpredictable task-switching and modality-shifting, the current study found reaction time costs associated with task-switching and modality-shifting. The patterns and the magnitude of either task-switching or modality-shifting costs were similar across all ages, as young children did not exhibit larger RT and accuracy costs than adults. The lack of a strong age effect on task-switching cost from preschool years is consistent with our previous finding using unisensory visual task-switching paradigm [44], as well as other developmental studies [43–46]. However, past studies, including our unisensory study, generally involved a long preparation window which may have obscured the age differences in the preparation component of task-switching. In the current study, advanced preparation was not encouraged as the task cue and the stimulus onset were synchronous. Even under this challenging condition, we did not observe any age interaction with either task-switching or modality-shifting costs. As the current study involved participants with a large age range, the lack of age interaction with any mode of attentional shift is particularly striking. The overall result illustrated that children as young as four were adept at switching attention between modalities and between tasks.

Although we found no differential age effect on the size of the attentional shift cost (for both task transition and modality transition), children were still slower and less accurate than adults. It is not clear what contributes to the overall lower performance in young children. One possibility is that different cognitive processes involved in task switching may have different timescales for maturation. For example, Weeda and colleagues [42] have suggested that processes that contribute to proactive interference from the competing task set may mature relatively early, whereas the preparation and top-down component may have a longer maturation timescale. It remains possible that although young children in our experiment did not exhibit greater within-subject performance costs by transition types, they were nonetheless more likely to be unprepared than adults for the upcoming task, resulting in lower overall accuracy and longer RT than observed in adults.

Our main interest was to investigate whether there are developmental differences in the facilitative or subadditive effects of cross-modal task-switching, through which we can glimpse into the organization of information processes in different age groups. Specifically, we hypothesised that younger children would experience a greater attentional bottleneck and show additive costs to shift attention between modality and task. This hypothesis was based on past findings reporting that younger children showed greater engagement with the frontal network when shifting attention across modalities, consequently suggesting a more effortful switch of attention through top-down mechanism [48]. In contrast, adults would be more likely to have a more mature segregated system to deal with information interference, and exhibit a cross-modal task-switching benefit as compared to the combined cost. To our surprise, we found no age interaction in the pattern of cross-modal task-switching cost. Furthermore, there was no evidence of a facilitative or a subadditive effect of cross-modal task-switching, in which modality-shift benefits task-switching, in any of the age groups. To our knowledge, this is the first study that reported an absence of benefit of simultaneous task and modality change.

Our result suggests that a common attentional resource between task-switching and modality-shifting is engaged during the current experimental paradigm. The result lends partial support to the previous studies that modality-shifting and task-switching are at least somewhat interdependent [25,56]. Although our result did not report a subadditive effect, it remains possible that parts of the processing pathways can be independent under different experimental context. One explanation for the disparate finding between the current and the past findings may be that the level of cost/benefit from a cross-modal event is dependent on the selection processes. If the benefit of modality-shift stems from the reduced interference among the task-associated attributes, it may not be surprising that the degree of benefit is also dependent on what selection processes are involved. A task that requires multiple and sequential selections may afford more opportunities for the cross-modal facilitative/ subadditive effect to emerge, since such a task is more likely to recruit multiple local networks. The current study is low in selection demands in either modality, task or response, as compared to other choice-based cross-modal task-switching studies with bimodal inputs, overlapping task attributes and responses. As a result, cross-modal facilitation may have little to act on to alleviate the already-small interference.

In task-switching studies, task transition has a cost-benefit relation with response repetition; response repetition benefits performance on task-repetition trials, but impedes performance on task-switch trials [7,57,58]. The phenomenon can be explained by episodic binding in the S-R event on the previous trial. When both response and task repeats, there is a priming facilitation from the previous S-R event. In contrast, when the task switches, the same response activates the irrelevant S-R event, creating a competition between the current and the previous S-R events [59]. The constraint by task transition on response repetition effect was also found in the current study, conforming to the episodic S-R binding account, as the benefit of response repetition was only evident on task-repetition trials, but not on task-switch trials (although, admittedly, we are unable to investigate response repetition cost on task-switch trials directly as it was confounded with task-switch costs).

In contrast, modality transition did not show the same constraint on response repetition. Response repetition showed benefits regardless of the modality transition. This indicates that modality is not an important constituent in the S-R event, or perhaps in the task representation itself, in the present study. Although a previous study on modality shift effects suggests that modality is a constituent of the S-R event [24], without a well-defined task that encompasses both auditory and visual modalities, it is not clear if the effect comes from modality-specific or task-specific components. Our result shows that, when the tasks are supramodal, modality information does not necessary form part of the episodic profile. This also implies that the processing cost associated with modality-shifting is likely to differ from the costs associated with task-switching, as modality-shifting is unlikely to change the whole S-R event as task-switching does. Importantly, both young children and adults showed similar interactions among response transition, modality transition and task transition. This indicates that both children and adults processed task, modality and response information in a comparable manner, formed similar mental representation of the task context, and are likely to engage the same cognitive operations in cross-modal task-switching tasks.
